# Intraoperative hypothermia risk trajectories in patients undergoing video-assisted thoracoscopic surgery: a retrospective study

**DOI:** 10.3389/fmed.2026.1860218

**Published:** 2026-07-13

**Authors:** Rui Chen, Xiaomin Ma, Sha Luo, Jing Li, Liwen Xu

**Affiliations:** 1 Operating Room, The Central Hospital of Wuhan, Tongji Medical College, Huazhong University of Science and Technology, Wuhan, Hubei, China; 2 Nursing Department, The Central Hospital of Wuhan, Tongji Medical College, Huazhong University of Science and Technology, Wuhan, Hubei, China

**Keywords:** hypothermia, risk, temperature management, trajectory, video-assisted thoracic surgery

## Abstract

**Background:**

Intraoperative hypothermia is common in video-assisted thoracic surgery (VATS) and can impair postoperative recovery. Most existing studies rely on static cross-sectional analyses, lacking dynamic descriptions of temperature changes and interactive risk factors.

**Objective:**

To investigate intraoperative hypothermia trajectories in VATS patients and provide evidence for predictive temperature management.

**Methods:**

A retrospective analysis was performed on the medical records of 571 patients who underwent thoracoscopic surgeries and satisfied the inclusion criteria at a tertiary hospital between January 2022 and December 2023. The patients were categorized into three distinct temperature groups based on their intraoperative hypothermia status: the normothermic group (*n* = 138), the hypothermia recovery group (*n* = 136), and the hypothermia non-recovery group (*n* = 297). Univariate analysis and logistic regression were employed to identify factors influencing temperature variations. Local weighted regression analysis was conducted on continuous intraoperative temperature monitoring data using Python 3.13, and Matplotlib was utilized to plot the fitted mean curves representing the three temperature trajectories.

**Result:**

No significant differences were found in gender, age, surgery duration, anesthesia duration, Intraoperative blood loss, preoperative body temperature (*P* > 0.05). Multinomial logistic regression showed that each 500 mL increase in intraoperative infusion volume significantly increased the risk of being in the hypothermia-recovered group (OR = 1.570, *P* < 0.001) and the hypothermia-nonrecovered group (*OR* = 1.305, *P* = 0.007). Lower BMI was a risk factor only for the nonrecovered group (*OR* = 0.891, *P* = 0.003). BSA and urine volume were not significant. Three distinct trajectories were identified: Horizontal “∫” (normothermic), “√” (hypothermia recovery), and “L” (hypothermia non-recovery).

**Conclusion:**

Individualized perioperative temperature management for VATS patients should be guided by dynamic intraoperative temperature trajectories, infusion requirements, and BMI stratification. Patients with low BMI (< 18.5 kg/m^2^) may have better rewarming capacity once hypothermia develops, while overweight or obese patients (BMI >23.9 kg/m^2^) are at higher risk of entering a non-recovery trajectory. These findings provide a practical basis for stratified hypothermia prevention and lay the groundwork for further precision management research.

## Introduction

Video-assisted thoracoscopic surgery (VATS) has become a major minimally invasive strategy for the management of multiple thoracic disorders and is regarded as the first-line standard treatment in numerous clinical scenarios (1). Compared with conventional thoracotomy, VATS is associated with less postoperative pain, shorter hospital stay, and faster postoperative recovery, thereby significantly improving perioperative quality of life (2). Despite these advantages, perioperative care for patients undergoing VATS remains challenging, and intraoperative hypothermia represents a common yet underrecognized complication (3). Intraoperative hypothermia is defined as a core body temperature below 36°C during surgery (4). Reportedly, the incidence of hypothermia in patients undergoing thoracic surgery under general anesthesia can be as high as 78.3% (3). This high prevalence is largely attributed to prolonged exposure of the thoracic cavity to the operating room laminar airflow, the application of cold irrigation solutions, and the suppression of thermoregulatory mechanisms by anesthetic agents, all of which render VATS patients particularly vulnerable to intraoperative hypothermia (5). The occurrence of hypothermia may lead to coagulation dysfunction (6), prolonged emergence from anesthesia (7), postoperative shivering (8), arrhythmias (9), an increased risk of surgical site infection (10), aggravated patient discomfort, and elevated medical expenses. Moreover, relevant studies have shown that persistent hypothermia may exacerbate delayed postoperative recovery (11), ischemia-reperfusion injury, and mucosal barrier dysfunction (12). Therefore, effective prevention and targeted management of intraoperative hypothermia are essential to optimize perioperative outcomes in patients undergoing VATS.

Existing studies on hypothermia in VATS primarily focus on its risk factors (13, 14), risk prediction models (5, 15), and the efficacy of intraoperative warming interventions (16). Nevertheless, these studies present several limitations. Traditional risk assessment typically conceptualizes hypothermia risk as a static outcome, with most employing cross-sectional designs that inadequately capture the dynamic evolution of core body temperature intraoperatively. Hypothermia risk prediction is often limited to a single time point, rarely enabling dynamic, prospective adjustment of warming strategies based on real-time temperature changes. In contrast, emerging evidence from other clinical contexts supports the value of dynamic physiological parameters for real-time risk stratification. For instance, Lou et al. (17) demonstrated that a dynamic metabolic indicator—the serum glucose-potassium ratio—was strongly associated with short- and long-term mortality in septic patients, highlighting the prognostic importance of time-varying biomarkers. In clinical practice, perioperative temperature management largely relies on nurses' empirical judgment or uniform warming protocols applied to all patients (7, 18). However, clinical evidence indicates that body temperature in VATS patients fluctuates dynamically throughout the procedure in association with surgical and anesthetic duration (19). Characterizing these distinct temperature trajectories is critical for developing targeted, predictive warming strategies. The conventional “one-size-fits-all” approach may be suboptimal, as it overlooks substantial interindividual variability in thermal dynamics. Accordingly, identifying intraoperative temperature trajectories in patients undergoing VATS can assist surgeons, anesthesiologists, and operating room nurses in recognizing heterogeneous thermal patterns, implementing predictive temperature management, and ultimately enhancing the quality and efficiency of perioperative thermal care.

This study was designed to address the insufficient characterization of intraoperative body temperature risk trajectories in patients undergoing VATS. The purpose of this study was to identify distinct trajectory patterns of intraoperative core temperature and to explore their associated influencing factors. We aim to provide evidence-based implications for the development of targeted and predictive thermal management strategies, thereby reducing the incidence of intraoperative hypothermia and optimizing overall perioperative outcomes in VATS patients.

## Methods

### Study design

Retrospectively enrolled patients undergoing VATS at a tertiary hospital in Wuhan from January 2022 to December 2023, and collected their medical records and surgery-related data. Inclusion criteria were as follows: (1) meeting the clinical indications for thoracoscopic surgery (20); (2) patients undergoing elective and uncomplicated video-assisted thoracic surgery; (3) being eligible for placement of a nasopharyngeal temperature probe; (4) having complete, continuous and valid intraoperative nasopharyngeal temperature records. Exclusion criteria were as follows: (1) complicated with severe underlying diseases that affect body temperature regulation; (2) conversion from VATS to open thoracotomy during the operation; (3) undergoing other major surgeries in addition to VATS; (4) experiencing massive intraoperative hemorrhage, requiring rapid and large-volume transfusion of unwarmed fluids or blood products, or developing severe circulatory instability during surgery; (5) incomplete medical records.

### Ethical consideration

This study followed the STROBE (Strengthening the Reporting of Observational Studies in Epidemiology) reporting guideline for observational studies and was performed in accordance with the Declaration of Helsinki. Ethical approval was granted by the Ethics Committee of Hospital (Approval Number: WHZXKYL2024-011-01). The requirement for informed consent was waived due to the retrospective nature of the study and the use of anonymized data.

### Temperature control

For patients undergoing VATS, the ambient temperature in the operating room was uniformly set at 22~24 °C with an indoor temperature fluctuation within ±1 °C. An inflatable warming blanket was routinely applied to cover the patients' lower limbs below the waist, starting from admission to the operating room until the end of surgery, with a constant temperature setting of 38 °C. All intraoperative infused fluids and irrigation fluids were prewarmed to 37 °C. This study aimed to explore the dynamic changes in intraoperative body temperature trajectories of patients under standardized external temperature management.

### Body temperature monitoring

All body temperature data in this study were collected *via* nasopharyngeal temperature monitors. After anesthesia induction, the temperature sensor probe was inserted into the patient's nasopharynx at a depth equivalent to the distance from the nasal ala to the ipsilateral mandibular angle (21). Intraoperative nasopharyngeal temperature was monitored every 5 min until the completion of surgery, and all temperature data were recorded and stored in the anesthesia information system.

### Influence factor

Based on literature review and expert recommendations (3, 5, 13, 14, 19), a total of ten potential risk factors influencing intraoperative body temperature changes were screened and identified in this study, including gender, age, body mass index (BMI), body surface area (BSA), preoperative body temperature, operative duration, anesthesia duration, intraoperative infusion volume, intraoperative blood loss, and urine volume. All the above indicators were extracted from the hospital electronic medical record system and anesthesia information system.

### Sample

A retrospective study design was adopted. The sample size was calculated based on an estimated incidence of intraoperative hypothermia of 78.3% in thoracic surgery. With a two-sided type I error of 0.05 and a marginal error of 5%, the minimum required sample size was determined to be 262 patients using the normal approximation formula for proportions (22). Considering an expected 20% rate of data loss or exclusion, at least 328 patients were required to be enrolled in this retrospective study. A total of 864 patients undergoing VATS were initially retrieved from the database. Among them, 182 patients did not meet the inclusion criteria, and 111 patients had discontinuous or invalid body temperature data. To improve the precision of estimates and to enable robust subgroup and trajectory analyses, all eligible consecutive patients who met the inclusion criteria during the study period were enrolled. This approach resulted in a final sample of 571 patients, which exceeds the minimum requirement but does not violate any statistical assumptions. The larger sample size enhances the stability of the fitted LOWESS trajectories and the reliability of the logistic regression estimates.

### Statistical analysis

Microsoft Excel was used to collect clinical data and continuous intraoperative body temperature data of patients undergoing VATS. Missing values were identified and quantified for all candidate variables. For continuous variables with a low missing rate (< 5%), missing data were replaced by the mean or median of observed values.

Based on retrospectively obtained intraoperative core body temperature monitoring data from VATS patients, all patients were classified into three groups according to the clinical criterion of core body temperature < 36 °C as hypothermia: (1) normothermic group (*n* = 138)—patients whose temperature remained ≥36.0 °C throughout the surgery; (2) hypothermia recovery group (*n* = 136)—patients whose temperature dropped below 36.0 °C at any point but subsequently returned to ≥36.0 °C before the end of surgery; and (3) hypothermia non-recovery group (*n* = 297)—patients whose temperature dropped below 36.0 °C and never recovered to ≥36.0 °C by the end of surgery.

To visualize the dynamic temperature trends, we performed locally weighted scatterplot smoothing (LOWESS) on the continuous intraoperative temperature data, with anesthesia time (minutes) on the horizontal axis and core body temperature (°C) on the vertical axis, implemented using Python 3.13 and the NumPy library. The analysis employed a Gaussian kernel function with a bandwidth parameter of τ = 10 min (corresponding to a characteristic distance of sqrt (τ) ≈ 14 min, approximately 3 data points). This value was determined based on the following considerations: (1) postoperative body temperature may fluctuate rapidly during the anesthesia recovery period, and a short time window enables sensitive capture of clinically relevant dynamic changes; and (2) a sensitivity analysis comparing fitting performance across τ ε {5, 10, 30, 50} min indicated that τ = 10 achieved the optimal balance between goodness-of-fit (RMSE) and clinical interpretability, whereas larger values would obscure the inflection point characteristics in the hypothermia recovery group. The fitted mean temperature curves for each group were plotted using the Matplotlib library (Python 3.13).

Statistical analyses were performed using SPSS 26.0 software. Continuous variables were expressed as mean ± standard deviation. One-way analysis of variance (*F*-test) was used for between-group comparisons if the data followed a normal distribution and homogeneity of variance. For skewed distribution data, variables were presented as median (interquartile range) [M (P_2_5, P75)], and the Mann-Whitney U test (*Z* value) was adopted. Categorical variables were described as number (percentage) [*n* (%)], and between-group comparisons were conducted using the chi-square (χ^2^) test. Variables with *P* < 0.05 in univariate analysis were included in the multinomial logistic regression model to identify independent influencing factors of body temperature trajectories. The odds ratio (*OR*) and its 95% confidence interval (*CI*) were calculated, statistical significance was defined as a *p*-value < 0.05.

## Results

### Sample characteristics

A total of 571 patients undergoing VATS were included in this study, of whom 296 were males (51.84%) and 275 were females (48.16%). The average age of patients in the three groups was 60.82 ± 10.74 years, and the incidence of intraoperative hypothermia was 75.83%. More sample characteristics are shown in Table 1.

**Table 1:** Comparison of basic patient information.

Variable	Normothermia group (*n* =138)	Hypothermia-recovered group (*n* = 136)	Hypothermia-nonrecovered group (*n* = 297)	Statistic	*P*-value
Age [yr, M(Q1,Q3)]	60.00 (53.75–67.25)	62.00 (55.00–68.00)	63.00 (55.00–69.00)	*Z* = 3.729	0.155
Intraoperative infusion volume [ml, M(Q_1_,Q_3_)]	1,500 (1,500–2,000)	2000 (1,500–2,000)	2000 (1,500–2,000)	*Z* = 17.347	< 0.001^*^
Intraoperative blood loss [ml, M(Q_1_,Q_3_)]	50 (20–110)	100 (32.50–110)	100 (20–110)	Z = 1.579	0.454
Urine volume [ml, M(Q_1_,Q_3_)]	600 (400–610)	610 (400–700)	610 (450–700)	*Z* = 9.063	0.011^*^
Preoperative body temperature [°C,M(Q_1_,Q_3_)]	36.5 (36.3–36.6)	36.5 (36.4–36.6)	36.5 (36.3–36.6)	*Z* = 5.492	0.064
BSA [m^2^, Mean ± SD]	1.70 ± 0.17	1.69 ± 0.22	1.65 ± 0.16	*F* = 4.795	0.009^*^
BMI [*n* (%)]				*χ^2^* = 19.047	0.001^*^
< 18.5 kg/m^2^	10 (7.25)	6 (4.41)	24 (8.08)		
18.5~23.9 kg/m^2^	58 (42.03)	68 (50.00)	180 (60.61)		
>23.9 kg/m^2^	70 (50.72)	62 (45.59)	93 (31.31)		
Gender [*n* (%)]				*χ^2^* = 4.405	0.111
Male	75 (54.35)	79 (58.09)	142 (47.81)		
Female	63 (45.65)	57 (41.91)	155 (52.19)		
Surgery duration [min, Mean ± SD]	141.00 ± 49.84	154.31 ± 42.39	142.73 ± 56.57	*F* = 2.880	0.057
Anesthesia duration [min, Mean ± SD]	238.78 ± 64.55	244.13 ± 47.20	236.70 ± 71.51	*F* = 0.614	0.541

BSA = 0.0061^*^height (cm) + 0.0124^*^weight (kg) – 0.0099. Differences between groups were analyzed by one-way analysis of variance (ANOVA, F-value) for normally distributed continuous variables, chi-square test (χ^2^) for categorical variables, and Z-test for non-normally distributed continuous variables. ^*^P-value < 0.05 was considered statistically significant.

### Univariate analysis of intraoperative hypothermia

A total of 571 VATS patients were divided into three groups according to the timing of intraoperative hypothermia occurrence: 138 cases in the normothermia group, 136 cases in the hypothermia-recovered group, and 297 cases in the persistent hypothermia-nonrecovered group. The results showed that there were statistically significant differences in intraoperative infusion volume, urine volume, BSA, and BMI (*P* < 0.05). However, there were no statistically significant differences in age, gender, operative duration, anesthesia duration, intraoperative blood loss, and preoperative body temperature (*P* > 0.05). More sample characteristics are shown in Table 1.

### Logistic regression analysis

Patient body temperature trajectory was set as the dependent variable. Based on univariate screening, intraoperative infusion volume, urine volume, BSA, and BMI were entered into a multinomial logistic regression model (23), with the normothermic group as the reference category. To improve clinical interpretability, infusion volume was re-scaled to 500-mL increments (i.e., volume in mL divided by 500) and treated as a continuous variable. The results are presented in Table 2. After this rescaling, each additional 500 mL of fluid was associated with a significantly increased risk of being in the hypothermia-recovered group (*OR* = 1.570, 95% *CI*: 1.258–1.959, *P* < 0.001) and the hypothermia-nonrecovered group (*OR* = 1.305, 95% *CI*: 1.077–1.581, *P* = 0.007). Lower BMI was a significant risk factor only for the hypothermia-nonrecovered group (*OR* = 0.891, 95% *CI*: 0.827–0.961, *P* = 0.003). BSA and urine volume showed no significant associations in either trajectory group (all *P* > 0.05). The interaction term between BMI and infusion volume (per 500 mL) was not statistically significant in either the hypothermia-recovered group (*OR* = 1.029, 95% *CI*: 0.962–1.102, *P* = 0.404) or the hypothermia-nonrecovered group (*OR* = 0.977, 95% *CI*: 0.919–1.038, *P* = 0.446), indicating no evidence of effect modification by BMI. More sample characteristics are shown in Table 2.

**Table 2:** Multinomial logistic regression analysis of independent factors associated with intraoperative body temperature trajectory in VATS patients.

Variable	Hypothermia-recovered group vs. normothermia group			Hypothermia-nonrecovered group vs. normothermia group		
	*OR* (95%*CI*)	Wald *χ^2^*	*P*-value	*OR* (95%*CI*)	Wald *χ^2^*	*P*-value
Intraoperative infusion volume (per 500 mL)^a^	1.570 (1.258–1.959)	15.960	< 0.001	1.305 (1.077–1.581)	7.357	0.007
BMI	0.979 (0.897–1.068)	0.226	0.634	0.891 (0.827–0.961)	9.072	0.003
BSA	0.781(0.149–4.084)	0.086	0.770	0.548 (0.136–2.208)	0.715	0.398
Urine volume	1.000(1.000–1.001)	0.885	0.347	1.000(1.000–1.001)	1.159	0.282
BMI × Intraoperative Infusion volume (per 500 mL)^b^	1.029 (0.962–1.102)	0.698	0.404	0.977 (0.919–1.038)	0.580	0.446

^a^Re-analyzed using a 500-mL increment to improve clinical interpretability; original analysis with 1-mL increment yielded OR = 1.001 (1.000–1.001) per mL, *p* < 0.001 and *p* = 0.012 respectively. ^b^Interaction term between BMI and Intraoperative infusion volume (per 500 mL). OR represents the multiplicative effect of the interaction.

### Analysis of intraoperative hypothermia trajectory

By analyzing the intraoperative body temperature monitoring data of 571 patients undergoing VATS, the patients were divided into three groups according to the occurrence of intraoperative hypothermia, as follows: Figure 2A shows the normothermia group, in which the core body temperature of patients remained above 36.0 °C throughout the entire operation. The body temperature began to decrease 30 min after the induction of anesthesia, entered a plateau phase between 90 and 180 min, maintained at around 36.4 °C, and then decreased slowly after 180 min but still remained above 36 °C. The narrow 95% CI indicated a high degree of consistency in temperature control within this group. Figure 2B shows the hypothermia-recovered group, where the body temperature of patients decreased rapidly to below 36 °C within 30 min after the induction of anesthesia, dropped to the lowest level of 35.8 °C at 60 min, then gradually increased, and recovered to above 36 °C at around 100 min. Figure 2C shows the hypothermia-nonrecovered group, in which patients developed hypothermia immediately after the induction of anesthesia, which dropped to 35.4 °C at 90 min, and the body temperature remained stable at 35.3–35.4 °C throughout the entire anesthesia period.

**Figure 1:**
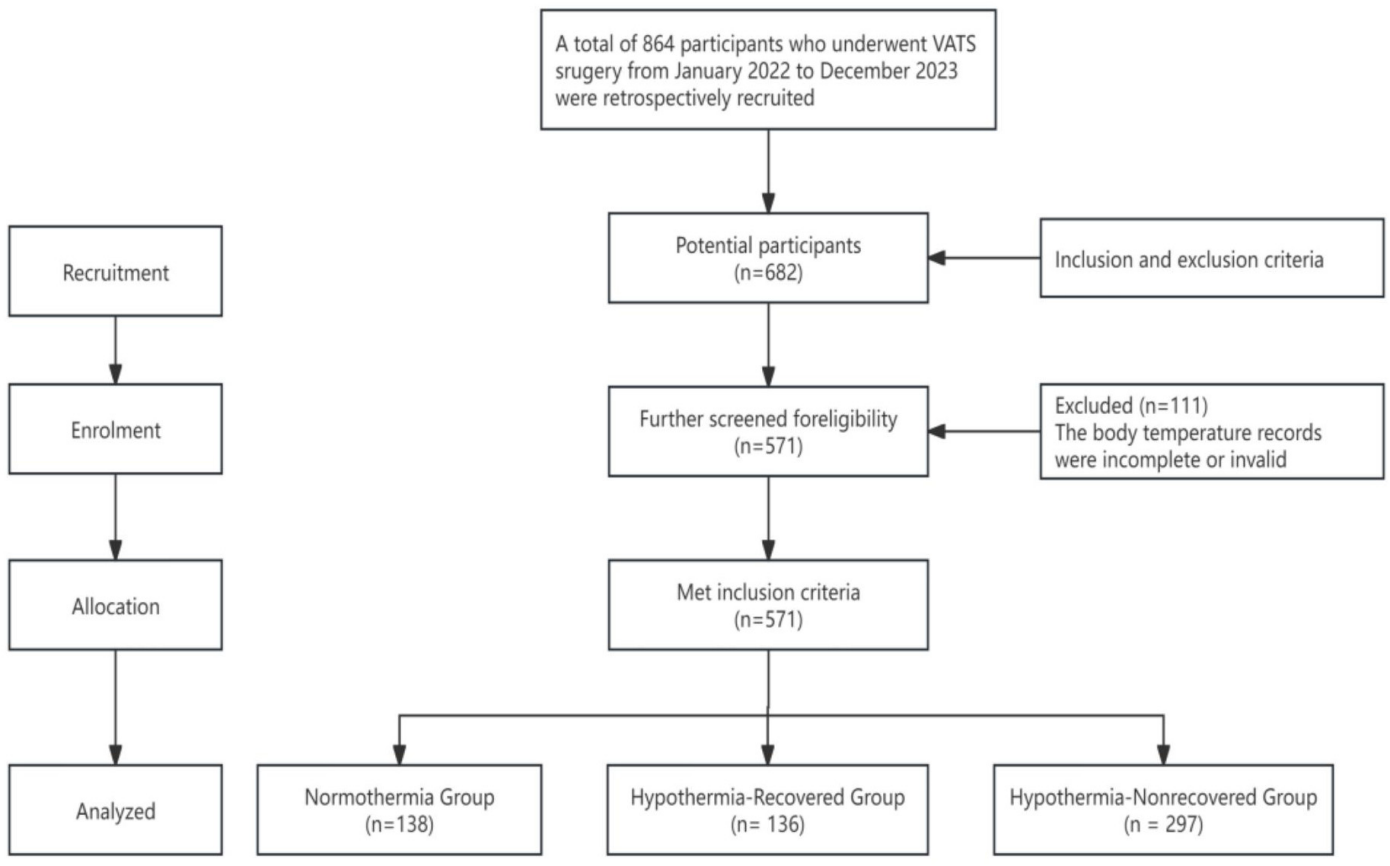
Flowchart of patient enrollment.

**Figure 2:**
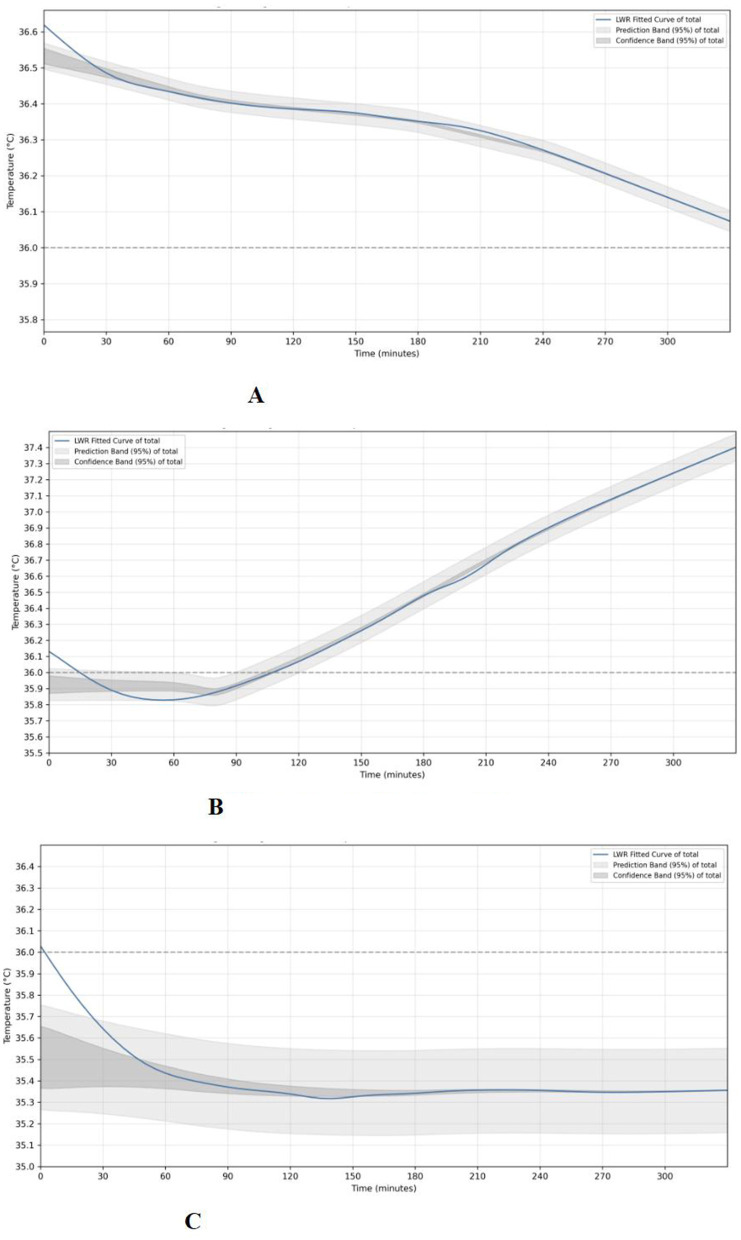
Intraoperative temperature trajectory of VATS patients. **(A)** represents the trajectory of the normothermia group, **(B)** represents the trajectory of the hypothermia-recovered group, and **(C)** represents the trajectory of the hypothermia-nonrecovered group. **(A–C)** are all locally weighted regression fitted curves, where the horizontal axis is anesthesia time (minutes) and the vertical axis is core body temperature (°C). The dark gray area indicates the 95% confidence band for the overall mean temperature trajectory (reflecting the uncertainty of the mean estimation), and the light gray area indicates the 95% prediction band for individual temperature observations (reflecting the fluctuation range of individual body temperature). The dashed line at 36.0 °C is the clinically commonly used critical value for intraoperative hypothermia.

Among the body temperature trajectories of the three groups, the normothermia group had the smallest body temperature fluctuation and the lowest risk of hypothermia; the hypothermia-recovered group had reversible hypothermia during anesthesia, while the hypothermia-nonrecovered group had the earliest occurrence and the longest duration of hypothermia events.

### The impact of intraoperative infusion volume on hypothermia trajectory

**Figure 3:**
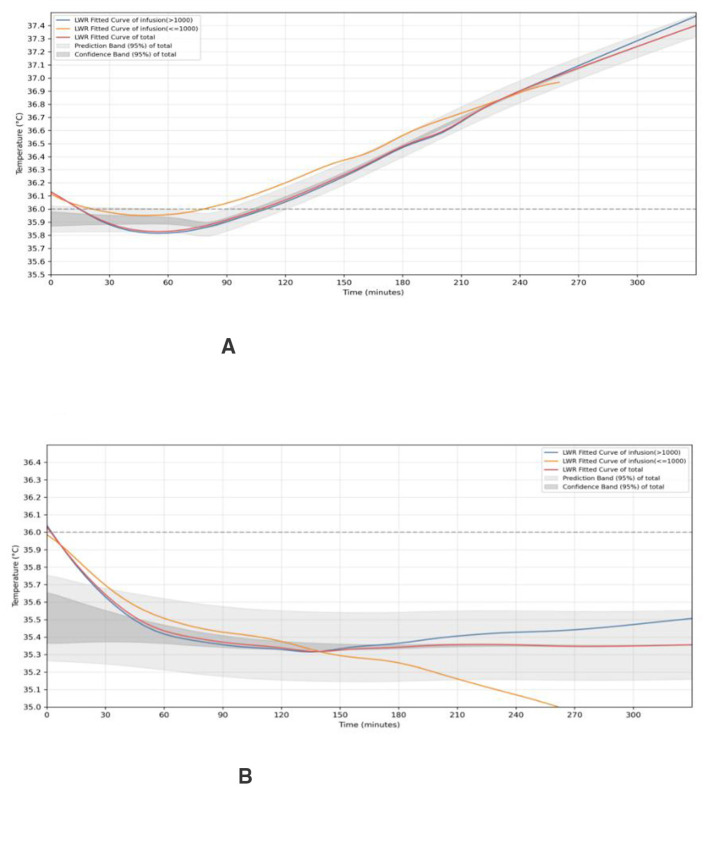
**(A)** Temperature trajectory patterns stratified by intraoperative infusion volume in VATS patients with hypothermia-recovered group. **(B)** Temperature trajectory patterns stratified by intraoperative infusion volume in VATS patients with hypothermia-nonrecovered group. The impact of intraoperative infusion volume on hypothermia trajectory. The blue curve represents the group with intraoperative infusion volume > 1,000 ml, the orange curve represents the group with intraoperative infusion volume ≤ 1,000 ml, and the red curve represents the overall fitted curve. The dark gray area indicates the 95% confidence band for the overall mean temperature trajectory, and the light gray area indicates the 95% prediction band for individual temperature observations.

Based on established clinical guidelines and expert consensus that intraoperative infusion volumes exceeding 1,000 mL significantly increase the risk of hypothermia (24), we dichotomized this variable into two groups: >1,000 mL and ≤ 1,000 mL for analysis. Figure 3 shows the changes in the trajectory of body temperature curves in patients with intraoperative hypothermia across different fluid volume groups. Figure 3A represents the hypothermia-recovered group: in patients with a fluid volume >1,000 ml, their body temperature decreased from 36.1 °C to the lowest point of 35.8 °C within 0–60 min, and the degree of hypothermia was associated with the high fluid volume. After 60 min, the body temperature recovered linearly, with a recovery slope significantly steeper than that in the low fluid volume group. For patients with a fluid volume ≤ 1,000 ml, their body temperature decreased from 36.1 to 35.9 °C within 0–60 min, with a slightly milder degree of hypothermia compared with the high fluid volume group. During 60–240 min, the body temperature gradually recovered, but the recovery rate was significantly slower than that in the high fluid volume group, and the recovery stagnated after 240 min. Figure 3B represents the hypothermia-nonrecovered group: in patients with a fluid volume >1,000 ml, their body temperature rapidly decreased from 36.0 °C to around 35.4 °C within 0–90 min, entering a hypothermic state. After 120 min, the body temperature gradually recovered, reaching 35.5 °C at 320 min. Although it did not return to the normal range, it showed a continuous improvement trend. For patients with a fluid volume ≤ 1,000 ml, their body temperature decreased from 36.0 to 35.5 °C within 0–90 min, with a slower decrease rate compared with the high fluid volume group. At approximately 140 min, the two curves intersected: patients in the low fluid volume group developed progressive hypothermia, while those in the high fluid volume group maintained a slow temperature recovery trend.

### The impact of BMI on the trajectory of hypothermia

**Figure 4:**
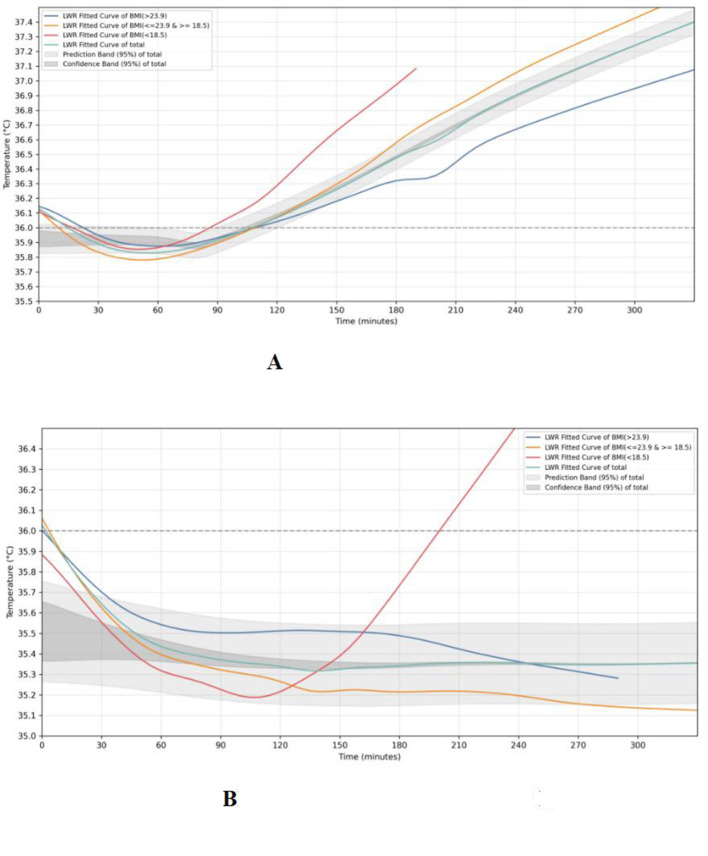
**(A)** Temperature trajectory patterns stratified by BMI in VATS patients with hypothermia-recovered group. **(B)** Temperature trajectory patterns stratified by BMI in VATS patients with hypothermia-nonrecovered group. The impact of BMI on the trajectory of hypothermia. The blue curve represents the subgroup with BMI > 23.9 kg/m^2^, the orange curve represents the subgroup with 18.5 kg/m^2^ ≤ BMI ≤ 23.9 kg/m^2^, and the red curve represents the subgroup with BMI < 18.5 kg/m^2^.The dark gray area indicates the 95% confidence band for the overall mean temperature trajectory, and the light gray area indicates the 95% prediction band for individual temperature observations.

Using a predefined BMI classification based on Chinese and Asian guidelines (24) (underweight: BMI < 18.5 kg/m^2^; normal weight: 18.5 kg/m^2^ ≤ BMI ≤ 23.9kg/m^2^; overweight/obese: BMI >23.9 kg/m^2^), we analyzed the impact of BMI on the trajectory of hypothermia. Figure 4 shows the changes in the body temperature curve trajectory of patients with intraoperative hypothermia in different BMI groups.

Figure 4A represents the hypothermia-recovered group, and the changes in body temperature of patients in each group are as follows: For patients with BMI < 18.5 kg/m^2^, the body temperature rapidly decreased from 36.1 to 35.9 °C within 30 min, and the degree of hypothermia was consistent with that of the other two groups; during 30–60 min, the body temperature further decreased to the lowest point of approximately 35.85 °C; after 60 min, the body temperature recovered at the fastest rate and with the largest amplitude, showing an explosive recovery, and returned to 36 °C at 90 min. For patients with 18.5 kg/m^2^ ≤ BMI ≤ 23.9 kg/m^2^, the body temperature decreased to the lowest point of 35.8 °C within 0–60 min, and rose to 36 °C at 110 min. For patients with BMI>23.9 kg/m^2^, the body temperature decreased more slowly than that of the other two groups within 0–60 min; it rose to 36 °C at 110 min, and after returning to normal, the rate of temperature recovery was slower than that of the other two groups. Figure 4B represents the hypothermia-nonrecovered group. For patients with BMI < 18.5 kg/m^2^, the body temperature rapidly decreased from 36.0 °C to the lowest point of 35.2 °C within approximately 0–100 min, with the largest degree of hypothermia; after 100 min, the body temperature continued to rise, exceeding 36.0 °C at 200 min, achieving hypothermia reversal. For patients with 18.5 kg/m^2^ ≤ BMI ≤ 23.9 kg/m^2^, the body temperature decreased to 35.2 °C at approximately 0–130 min, with the early decrease amplitude consistent with that of the low-weight group; after 130 min, the body temperature remained sluggish, maintained at the platform period of 35.1–35.2 °C, and showed persistent hypothermia throughout the process. For patients with BMI > 23.9 kg/m^2^, the body temperature decreased to 35.5 °C within 0–90 min, with the smallest degree of hypothermia; after 180 min, the body temperature slowly decreased, showing no recovery throughout the process and presenting persistent hypothermia.

## Discussion

Based on an in-depth analysis of intraoperative body temperature trajectories in patients undergoing VATS, this study confirmed that intraoperative body temperature is not a static indicator but a dynamic process following specific physiological patterns. Using a machine learning-based locally weighted regression model, three clinically distinct body temperature patterns were clearly identified: the normothermia group, the hypothermia-recovered group, and the hypothermia-nonrecovered group. The three types of temperature trajectories provide novel insights for refined intraoperative body temperature management.

In the normothermia group, the intraoperative body temperature curve presented a horizontal “∫” shape. According to the rate of temperature decline, the trajectory was divided into three phases: an initial rapid decline stage, a middle slow decline stage, and a terminal rapid decline stage. Although the overall body temperature showed a downward trend, it remained above 36 °C. As shown in Figure 2A, the core temperature dropped rapidly to approximately 36.4 °C within the first 90 min after anesthesia induction, with the maximum decline slope during this period. This phenomenon may be related to the imbalance of thermoregulatory function caused by anesthetics and surgical factors, leading to lower heat production than heat dissipation in the body (19), while the body temperature still remained within the normal range at this stage. During 90–180 min after anesthesia, the patients' body temperature entered a plateau phase, with the curve almost parallel to the X-axis and stabilized at around 36.4 °C, where heat production was roughly equivalent to heat loss. After 180 min of surgery, the decreasing rate of body temperature gradually accelerated, and the core temperature of most patients dropped to nearly 36 °C. With the extension of operative time, the thermoregulatory function of most patients entered a decompensatory period, and systemic heat dissipation was far greater than heat production. Without external intervention, a continuous decrease in body temperature would be predictable. For patients in this group, the core management focuses on maintaining temperature homeostasis during the plateau phase and preventing advanced thermoregulatory decompensation. Intraoperatively, core temperature is recommended to be recorded every 15 min, and the core-peripheral temperature gradient should be continuously monitored. Continuous heat preservation and temperature monitoring are also required in the post-anesthesia care unit after surgery to prevent the occurrence of late-stage thermoregulatory decompensation.

In the hypothermia-recovered group, the intraoperative body temperature curve presented a gentle “√” shape, which could be divided into a rapid decline phase and a gradual rewarming phase. As shown in Figure 2B, the body temperature in this group presented a trend of decreasing first and then rising, fluctuating between 36.5 and 37.3 °C. Within 60 min after the initiation of anesthesia, the core temperature dropped rapidly from the normal range to approximately 35.8 °C, resulting in a hypothermic state. After 60 min, body temperature began to rise gradually and recovered above 36 °C at about 100 min. This changing pattern may be explained as follows: in the early stage of anesthesia, anesthetics inhibit the function of the central thermoregulatory center. Combined with heat loss in the surgical environment, the body temperature decreases rapidly within 60 min. After 60 min, along with the metabolism of anesthetic drugs and the gradual reconstruction of the balance between body heat production and heat dissipation, the compensatory hypothermia regulatory mechanism is activated (25). Meanwhile, intraoperative warming interventions further facilitate the recovery of body temperature. For patients in this group, the core management priority lies in precise temperature control during the critical rewarming period. Continuous warming measures should be maintained throughout the rewarming stage from 60 to 120 min, with the core temperature controlled within 36~37.5 °C (26). When the temperature approaches or reaches 37.5 °C, active warming may be appropriately reduced or discontinued with close monitoring. Attention should be paid to the risk of over-warming. A core temperature exceeding 38 °C may induce adverse physiological reactions including tachycardia, sweating, vasodilation, and a 10%−13% increase in metabolic demand per °C (27). Therefore, intraoperative warming is not universally enhanced; maintaining body temperature within the physiological range is the fundamental principle. The physiological disturbances and potential risks caused by excessive warming also require high attention and proactive management from anesthesiologists and the surgical team.

In the hypothermia-nonrecovered group, the intraoperative body temperature curve presented a gentle “L” shape, which can be divided into a rapid decline phase and a sustained plateau phase. A preoperative core temperature ≤ 36 °C serves as a crucial early warning indicator for identifying high-risk patients in this group. As shown in Figure 2C, patients in this group had a relatively low preoperative body temperature, with an average of approximately 36 °C. Within 90 min after anesthesia initiation, their core temperature dropped sharply to around 35.4 °C. This abrupt temperature decrease may result from the inhibition of the hypothalamic thermoregulatory center and peripheral vasodilation induced by anesthetics, as well as a sharp reduction in heat production caused by muscle relaxants (28). In addition, the low-temperature operating room environment and evaporative heat loss due to body cavity exposure further exacerbate heat dissipation, leading to an imbalance of heat production < heat loss and consequently a drastic drop in body temperature (29). After 90 min, body temperature entered a prolonged plateau phase, fluctuating narrowly between 35.3 and 35.4 °C. At this stage, peripheral vasoconstriction reached its limit, the core-peripheral temperature gradient stabilized, and heat dissipation no longer increased. Nevertheless, major compensatory heat-generating mechanisms, including brown adipose tissue thermogenesis and shivering, remained suppressed by deep anesthesia and failed to effectively elevate heat production. A fragile balance of heat production = heat dissipation was therefore established, keeping body temperature persistently at a low level (24). For this patient group, the core management focus is targeted pre-warming before surgery and in the early anesthetic period. After patients enter the operating room, the room temperature should be adjusted to 22–24 °C to avoid cold stress initiation. Prior to anesthesia induction, it is recommended to apply forced-air warming blankets at 38 °C for pre-warming for more than 30 min, and active warming should be continued throughout tracheal intubation and other procedures to offset early anesthetic heat loss (30–32). It is recommended that all infused fluids and blood products be preheated to 37 °C before administration (33), and that intraoperative irrigation fluids be warmed to 38–40 °C (29). During irrigation, it is recommended to retain the fluid in the abdominal cavity for 3 min to ensure adequate heat absorption (34), thereby mitigating the sharp decline in intraoperative body temperature.

This study, using multinomial logistic regression (Table 2), identified significant associations of intraoperative infusion volume and BMI with intraoperative body temperature trajectory subgroups in patients undergoing VATS. In the comparison between the hypothermia-recovered group and the normothermia group, each 500 mL increase in intraoperative infusion volume was associated with an odds ratio of 1.570 (95% *CI*: 1.258–1.959, *P* < 0.001) for belonging to the hypothermia-recovered group. In the hypothermia-nonrecovered group vs. the normothermia group, the corresponding OR was 1.305 (95% *CI*: 1.077–1.581, *P* = 0.007). These findings indicate that larger infusion volume is associated with an increased risk of being classified into either hypothermic subgroup, regardless of eventual temperature recovery. Regarding BMI, a significant protective effect was observed only in the hypothermia-nonrecovered group (*OR* = 0.891, 95% *CI*: 0.827–0.961, *P* = 0.003), suggesting that higher BMI is associated with a lower risk of belonging to the nonrecovered subgroup. In the hypothermia-recovered group, BMI showed no significant effect (*P* = 0.634). BSA and urine volume were not statistically significant in either comparison.

Figure 4 further illustrates the dynamic changes in body temperature over time stratified by BMI. In the hypothermia-recovered group (Figure 4A), all BMI subgroups showed a temperature increase after reaching a nadir. Patients with BMI < 18.5 kg/m^2^ (red curve) experienced the greatest temperature drop (nadir approximately 35.9 °C), but subsequently demonstrated the fastest rewarming rate, with final temperature approaching normal levels. In contrast, patients with BMI > 23.9 kg/m^2^ (blue curve) had a more gradual decline and a smaller rewarming amplitude. In the hypothermia-nonrecovered group (Figure 4B), patients with BMI < 18.5 kg/m^2^ reached a nadir of approximately 35.2 °C at around 120–150 min and then showed a slow rewarming trend, indicating some endogenous thermoregulatory recovery capacity. Conversely, patients with BMI ≥ 18.5 kg/m^2^ (blue and orange curves) exhibited a continuous downward or plateauing trend without clear rewarming. These results challenge the traditional notion that higher body fat provides better heat preservation: under hypothermic conditions, underweight patients demonstrated stronger temperature recovery capacity, whereas overweight or obese patients may have impaired heat conduction due to the insulating layer of adipose tissue or blunted thermoregulatory responses, leading to difficulty in rewarming. Such nonlinear, context-dependent risk modulation has also been observed in other clinical settings; for instance, Lou et al. (35) reported a J-shaped association between the neutrophil-to-PNI ratio and mortality in septic patients. Taken together, the results from Table 2 and Figure 4 suggest that perioperative temperature management in VATS patients should not rely on a single clinical indicator. Although increased intraoperative infusion volume is associated with a higher risk of hypothermia, the effect of BMI on temperature trajectory is subgroup-specific. Patients with low BMI may still recover after developing hypothermia, whereas once patients with high BMI enter a nonrecovered trajectory, reversal becomes difficult. Therefore, individualized temperature management strategies based on dynamic intraoperative temperature trajectories, BMI stratification, and fluid requirements should be adopted to reduce the risk of hypothermia-related complications.

## Study limitations

This study was based on a retrospective data analysis. Although body temperature records were automatically collected by the monitoring system, the use of nasopharyngeal temperature probes is subject to structural constraints and clinical operational variations (e.g., inconsistent insertion depth, surgical interference), which may introduce fluctuations and confounding bias. Additionally, due to limitations in data collection and integrity, other potential risk factors for intraoperative hypothermia in VATS patients were not analyzed, warranting future studies with a broader range of covariates. Of note, the large sample size provided high statistical power; therefore, clinical relevance was assessed based on the magnitude of odds ratios (ORs) and their confidence intervals rather than *p*-values alone. Finally, this study fitted body temperature curves and analyzed temperature trajectories using continuous data but did not achieve individual risk prediction for single patients, which will be a key focus of our subsequent research.

## Conclusion

This study identified three characteristic intraoperative hypothermia trajectory patterns in patients undergoing video-assisted thoracic surgery (VATS) and revealed the dynamic interaction between thermoregulatory failure and compensatory mechanisms. The distinct temperature trajectories of the normothermia group, the hypothermia-recoveredgroup, and the hypothermia-nonrecovered group provide new insights into predictive temperature management. They enable clinical nursing staff to implement targeted and timed early interventions during critical time windows, so as to prevent the occurrence of intraoperative hypothermia. This study also establishes a trajectory-guided framework for intraoperative body temperature management. Future research should further explore a dynamic temperature gradient evaluation system, an intelligent early warning system, and an artificial intelligence-driven real-time decision support system based on the body temperature trajectory model. Ultimately, these efforts will promote the individualized and precise upgrading of perioperative temperature management.

## Data Availability

The data analyzed in this study is subject to the following licenses/restrictions: The data set related to this study can be accessed from the corresponding author upon reasonable request. Requests to access these datasets should be directed to Liwen Xu 874854918@qq.com.
